# Eating Pattern, Rumen Fermentation, and Microbial Population in Dairy Cows Exhibiting Divergent Methane to Carbon Dioxide Ratio in Breath

**DOI:** 10.1111/asj.70123

**Published:** 2025-10-29

**Authors:** R‐Jun Frederick Gaspe, Taketo Obitsu, Shuhei Takizawa, Miho Fujimori, Takumi Shinkai, Toshihisa Sugino

**Affiliations:** ^1^ Graduate School of Integrated Sciences for Life Hiroshima University Higashi‐Hiroshima Japan; ^2^ Agriculture Department Capiz State University Pilar Capiz Philippines; ^3^ Institute of Livestock and Grassland Science National Agriculture and Food Research Organization Tsukuba Ibaraki Japan

**Keywords:** body weight, eating behavior, methane production, propionic acid, rumen

## Abstract

This study aimed to elucidate the association of methane (CH_4_) production with eating behavior, rumen fermentation, and the rumen microbial population among dairy cows with varying CH_4_ to carbon dioxide (CO_2_) ratios in their breath, as measured by the sniffer method. Eighteen lactating Holstein cows were divided into three groups: low (LEm), mid (MEm), and high (HEm) CH_4_:CO_2_ ratios. Estimated CH_4_ production was lower in the LEm group than in the HEm group. Daily dry matter intake and milk production did not differ among the groups, whereas body weight was lower in the LEm group than in the MEm group. Eating time tended to be longer at night for the LEm group than for the HEm group. The ruminal molar proportion of acetate was lower, whereas that of propionate was higher in the LEm group than in the HEm group. Additionally, bacteria producing lactate and succinate were more prevalent in the LEm group, which may be associated with the higher ruminal propionate and lower estimated CH_4_ production in the LEm group. These findings suggest that body weight and eating time may be linked to variations in CH_4_:CO_2_ ratios in the respiration gases of cows that have the distinct rumen microbial community.

## 1 Introduction

Methane (CH_4_) is a potent greenhouse gas that contributes significantly to atmospheric warming and climate change, with a global warming potential of 28 times higher than carbon dioxide (CO_2_). Agricultural practices, including ruminant enteric fermentation and manure management, account for 41% of global CH_4_ emissions (Saunois et al. 2016). Specifically, enteric CH_4_ emissions from dairy cows represent 4% of total anthropogenic CH_4_ emissions globally (Wu et al. 2018). Although dairy cattle contribute only a small portion to overall greenhouse gas emissions, the industry has faced scrutiny, prompting increased awareness of its environmental footprint, which leads to consumer pressure for improved efficiency and sustainability practices, as well as to investigate mitigation strategies to lessen its environmental impact (Kamalanathan et al. 2023).

Several options exist to mitigate enteric CH_4_ emissions from dairy cows (Beauchemin et al. 2022), with genetic selection emerging as a viable and appealing approach (Kamalanathan et al. 2023). Utilizing phenotypic traits to identify high‐ and low‐CH_4_‐emitting cows is crucial for guiding future genetic selection efforts to reduce CH_4_ emissions in the dairy industry. Developing a practical and cost‐efficient measurement protocol is imperative to acquire phenotypic data of CH_4_‐related traits, encompassing daily CH_4_ production, CH_4_ yield, and intensity (Stepanchenko et al. 2023). A recent study by Uemoto et al. (2024) suggests that the ratio of CH_4_ to CO_2_ in respiration breath collected from the automatic milking system (AMS) using spot gas sampling (sniffer method) may serve as an indicator and can be utilized to select dairy cows with low CH_4_ emissions without compromising their productivity. Furthermore, using the breath CH_4_:CO_2_ ratio measured by the sniffer method as an independent variable, empirical multiple regression equations have been developed to easily estimate CH_4_ production of dairy cows in practical farm conditions (Suzuki et al. 2021). The validity of the estimation of CH_4_ production by the sniffer method could be revealed by simultaneously comparing the estimated CH_4_ production trait with several physiological and microbial traits affecting CH_4_ production such as profiles of rumen fermentation, microbial community, milk components, and feeding behavior in cows exhibiting divergent CH_4_ production.

Regarding the variation in CH_4_ production levels among individual animals, microbial analysis studies have revealed distinct populations of bacteria and archaea in the rumen of low‐ and high‐CH_4_‐emitting dairy cows (Stepanchenko et al. 2023). However, the factors driving this variation in microbial communities and CH_4_ emissions remain unclear. Recent research suggests that feeding behavior in dairy cows is heritable (Cavani et al. 2022). This raises the possibility that specific feeding behavior traits, such as diurnal eating patterns, may be associated with variations in both rumen microbial populations and CH_4_ emissions (Benchaar and Hassanat 2020; De La Guardia‐Hidrogo and Paz 2021). In addition, daily rumination time has also indicated the link to CH_4_ production (Mikuła et al. 2022).

Furthermore, variations in feeding time and rumination time can influence salivary secretion. Longer chewing time leads to increased saliva production (Beauchemin 2018). Saliva plays a crucial role in maintaining rumen pH and secretes antibacterial compounds like lysozyme, immunoglobulin A, and antibacterial peptides (Akula et al. 2023). Not only the quantity of saliva secretion but also the quality of saliva, such as the concentration of antibacterial compounds, may affect the microbial community in the rumen (Palma‐Hidalgo et al. 2021). Therefore, variations in feeding behavior may indirectly affect rumen microbes and CH_4_ emissions through the buffering and antimicrobial properties of saliva.

Hence, this study aimed to elucidate the variation in milk production, rumen fermentation, CH_4_ production, and feeding and rumination behavior, as well as rumen microbial population among cows exhibiting high, mid, and low CH_4_:CO_2_ ratios in their breath. We hypothesize that dairy cows could be categorized as low or high CH_4_ emitters based on the breath CH_4_:CO_2_ ratio measured by the sniffer method. The low emitters will exhibit distinct rumen microbial populations characterized by lower levels of methanogenic microorganisms, leading to altered rumen fermentation dynamics associated with lowered CH_4_ production, which may be linked with altered feeding behavior patterns and/or components in secreted saliva.

## 2 Materials and Methods

### 2.1 Animals, Diets, and Experimental Design

This experiment was carried out according to the guidelines specified by the Animal Care and Use Committee of Hiroshima University. The committee approved the procedures in this study (No. E21‐1).

The experiment was carried out in a free‐stall barn equipped with an individual door feeder (RIC system, HokoFarm Group, Emmelord, the Netherlands) and AMS (VMSV300, DeLaval, Tumba, Sweden). Eighteen lactating Holstein dairy cows (BW = 652 ± 15.0 kg, parity = 2.0, days in milk = 182 ± 25.4) were individually assigned to each feeder. Twelve out of 18 feeders were equipped to measure and record the feed weight in the feed trough at the time of opening and closing the feeder gate. The remaining six feeders recorded only the times of opening and closing the gate without recording the feed weight. The experiment lasted 21 days, with the first 2 weeks serving as an adjustment period and 1 week of data collection.

For 21 days, cows were provided with a partial mixed ration (PMR) composed of 12% dehydrated corn silage, 22% Italian ryegrass silage, 10% oats hay, 13% alfalfa hay, and 42% formula feed on a dry matter (DM) basis (Table 1). The PMR was delivered three times daily (10:00, 13:00, and 15:30 h) into a feed trough. The total amount of PMR was provided to fulfill the TDN requirement of lactating dairy cows based on the Japanese feeding standard for dairy cows (NARO 2017). Additionally, cows were also provided with commercial concentrate at each milking in the AMS according to the milk yield of each cow (3–8 kg/day). Even if cows entered AMS during a time when milking was not permitted, small portions of the concentrate diet were provided for 2 min (0.5 kg/min). Water and mineral blocks were freely accessed by the animals throughout the experiment. Cows were milked by AMS, where the milk yield and milking time for each cow were automatically recorded upon milking every day.

**TABLE 1:** Ingredients and chemical composition of partial mixed ration (PMR) and concentrate provided at milking robot.

Item	PMR	Concentrate
Ingredients, % DM		
Dehydrated corn silage	11.5	
Italian ryegrass silage	22.1	
Oats hay	9.9	
Alfalfa hay	12.8	
Formula feed^a^	41.6	
Calcium carbonate	0.47	
Vitamin mix	0.65	
Sodium bicarbonate	0.46	
Mold absorbent	0.22	
Salt	0.3	
Nutrient composition, %DM		
Dry matter (% fresh matter)	61.9	89.9
Crude ash	14.0	8.9
Crude protein	14.0	17.3
Neutral detergent fiber (aNDFom)	36.8	20.5
Ether extract	4.72	2.70
Nonfiber carbohydrate (NFC)	30.5	50.7
Estimated TDN^b^	69.0	85.7

^a^ Formula feed comprised flaked corn, rolled barley, soybean meal, heated soybean, cottonseed, wheat bran, corn gluten meal, beet pulp, soybean hull, calcium carbonate, and calcium phosphate.

^b^ TDN content was estimated based on the Standard Feed Table of NARO (2009).

### 2.2 Sampling and Measurement

#### 2.2.1 Intake, Eating, and Rumination Behavior

Daily PMR intake and visiting time for 12 cows assigned to feeders with weight recording were obtained by summing the recorded data of the RIC system for the amount of feed consumed and time duration at each visit. For cows assigned to feeders without weight recording (*n* = 6), only visiting time duration at the feeders was summed in the same manner as described above, whereas their daily PMR intake was estimated by subtracting the amount of feed remaining recorded manually from the amount of daily feed offered. The samples of offered PMR were collected every morning and dried at 70°C in an air‐forced oven for 72 h to measure DM content. The dry matter intake (DMI) of PMR was calculated based on the recorded feed intake and DM content of the PMR offered each day. Furthermore, diurnal variation of PMR intake (*n* = 12) and visiting time to feed trough (*n* = 18) were calculated by summarizing the data across specific 4‐h intervals throughout the day: from 10:00 to 14:00 h (encompassing the first to second delivery of PMR), from 14:00 to 18:00 h (corresponding to the time frame after the second delivery to the third delivery of PMR); 02:00 to 06:00, and 06:00 to 10:00 h; and from 02:00 to 06:00, 06:00 to 10:00. Visiting time duration from entry until leaving the feed trough was used as a proximate of eating time of PMR. Daily intake of concentrate diet at AMS was assumed to be the same amount as the record provided by the AMS. Daily DMI of PMR, concentrate, and total diet (PMR plus concentrate) were averaged for 7 days from Days 15 to 21. Rumination behavior was monitored by a motion sensor (U‐motion, Desamis Co. Ltd., Tokyo, Japan) attached to the neck collar of experimental cows, and the 7‐day average from Days 15 to 21 of daily rumination time recorded by the sensor system was regarded as the time spent rumination.

#### 2.2.2 Body Weight and Milk Sample

The live body weight (LW) of cows was measured before the second delivery of PMR on Day 20 of the experiment. Individual milk yield was collected from the stored data in the AMS and averaged for 7 days from Days 15 to 21. Milk samples collected on Days 19–21 were used to measure milk components using near‐infrared spectrometry at an analytical center (Kinki‐dairy Farmer Coop, Ono‐shi, Japan). Energy‐corrected milk (ECM) was calculated according to the equation described by Tyrrell and Reid (1965);

$$\begin{array}{c} \mathrm{ECM}\;\left(\mathrm{kg}/\mathrm{day}\right)=\text{milk yield}\;\left(\mathrm{kg}/\mathrm{day}\right)\\ \times \left(376\times \text{milk}\;\mathrm{fat}\;\left[\%\right]+209\times \text{milk protein}\;\left[\%\right]+948\right)/3138. \end{array}$$

#### 2.2.3 Methane Production

For estimating daily CH_4_ production, the concentration of CH_4_ and CO_2_ in the breath of individual cows during milking in the AMS was analyzed for 7 days from Days 15 to 21 by a sniffer method (Suzuki et al. 2021) with a Microportable Greenhouse Gas Analyzer (Model: 909‐0050, ABB/LGR, San Jose, CA, USA). Briefly, the air around the feed bin in the AMS was vacuumed with the pump (approximately 6 L/min). Two gas inlets covered with mesh filters were placed near the outlet of a feed hopper. The two inlet lines were combined into one gas line fitted with an air filter, a flow meter, a small mixing chamber, and an air pump. The gas from the mixing chamber entered the gas analyzer. The peak gas concentrations of CO_2_ at milking were adjusted to approximately 3000–10,000 ppm with the position of the gas inlets. The gas concentrations were measured continuously for 7 days during the experimental period. The CH_4_:CO_2_ concentration ratio was calculated using the average CH_4_ and CO_2_ concentration during the milking of each cow, with correcting background gas concentration measured during the nonmilking period (Oikawa et al. 2022). The averaged ratios and average ECM for 7 days and LW were applied to the prediction equations (Suzuki et al. 2021) to estimate daily CH_4_ production (L/day) as follows:

$${\mathrm{CH}}_{4}\;\left(L/\mathrm{day}\right)=-507+0.536\;\mathrm{LW}+8.76\;\mathrm{ECM}+5029\;{\mathrm{CH}}_{4}:{\mathrm{CO}}_{2}.$$

The methane conversion factor (MCF, J/100 J), which represents the proportion of methane energy to dietary gross energy, was also estimated using the following equations described by Suzuki et al. (2021):

$$\mathrm{MCF}\;\left(J/100\;J\right)=2.91-0.0498\;\mathrm{ECM}+51.0\;{\mathrm{CH}}_{4}:{\mathrm{CO}}_{2}.$$

#### 2.2.4 Rumen Fluid and Saliva

On Day 20 of the experiment, rumen fluid was collected using a stomach tube at 3 h after the first delivery of PMR (13:00 h). Immediately after collection, oxidation and reduction potential (ORP) and rumen pH were measured using a digital pH meter (LAQUA Horiba D‐210P; LAQUA Horiba pH/ION meter F‐27, HORIBA Advanced Techno Co. Ltd., Kyoto, Japan) with a glass electrode. Within 30 min after collection, the rumen fluid was filtered with a four‐layer gauze, and the filtrate was stored at −30°C for volatile fatty acid (VFA) analysis. Immediately after rumen fluid collection, a saliva sample from individual cows was also collected from the oral cavity by inserting a blade hose (TOYOX SUPER, 8 × 13 mm TOYOX CO. LTD, Toyama, Japan) and suction using an air pump (DA‐40S, ULVAC KIKO Inc., Miyazaki, Japan). The pH of saliva was measured similarly to rumen pH, and the collected saliva was stored in a freezer at −30°C until the in vitro study.

### 2.3 In Vitro Incubation With Saliva From Individual Cows

At 6 months after the in vivo experiment in the barn, an in vitro experiment was conducted to assess the impact of saliva quality from individual cows on CH_4_ production and rumen fermentation. Defrosted saliva from each animal was separately filtered with a four‐layer gauze and centrifuged at 16,300 × *g* for 5 min (Palma‐Hidalgo et al. 2021). Rumen fluid was collected from two cows selected from the same group used in the in vivo study, and these cows were fed a similar PMR to that used during the saliva collection period.

For the in vitro incubation, the inoculum was prepared by adding 2 mL of filtered mixed rumen fluid collected from the cows and 4 mL of prewarmed McDougall's buffer solution or prepared saliva. This mixture was then transferred into duplicated 10‐mL serum bottles, each containing 0.05 g of the dried PMR sample used in the in vivo study. The empty bottles without the PMR sample were treated in the same manner for blank incubation of each inoculum. The bottles were then flushed with nitrogen gas for 10 s to remove air, sealed with butyl rubber stoppers and aluminum crimps, and placed in a water bath at 39°C with a shaking speed of 60 shakes per minute for 24 h. After the incubation for 24 h, the total volume of gas produced during incubation was measured by puncture using a 10‐mL glass syringe with a 21‐G needle, and a portion of the gas was transferred into evacuated tubes for short‐term storage till CH_4_ analysis. A set of incubations was repeated three times on three separate days.

### 2.4 Chemical Analysis

Dried and ground samples of PMR and concentrate were analyzed for DM, crude protein (CP), ether extract (EE), crude ash, and neutral detergent fiber (aNDF_OM_) (AOAC 1999; Van Soest et al. 1991). The concentration of VFA in the rumen fluid and inoculum after in vitro incubation was analyzed by gas chromatography with a flame‐ionized detector (GC2014, Shimadzu, Kyoto, Japan) with a BP‐21 column (25 m length × 0.53 mm diameter, 0.5 μm in thickness, SGE Analytical Science, Australia). Methane concentration in gas produced in vitro was analyzed by gas chromatography with a flame ion detector (GC‐7A, Shimadzu) with a stainless column (2 m × 2 mm) packed with molecular sieve 5A.

### 2.5 Microbial Population Analysis

#### 2.5.1 Amplicon Sequencing

DNA extraction was conducted according to the previous report (Takizawa et al. 2023). Total DNA was extracted from 500 μL of rumen fluid using the FastDNA SPIN Kit for Soil (MP Biomedicals, Santa Ana, CA, USA), according to the manufacturer's instructions. DNA was quantified using the Qubit dsDNA BR Assay Kit (Life Technologies, Carlsbad, CA, USA), and all DNA samples were diluted to a concentration of 5 ng/μL with nuclease‐free water. Amplicon sequencing was performed according to the previous report (Takizawa et al. 2023) with some modifications. DNA libraries were constructed using two primer sets for the bacterial 16S rRNA V3–V4 regions (341F, 5′‐CCTACGGGNGGCWGCAG‐3′; 805R, 5′‐GACTACHVGGGTATCTAATCC‐3′) and archaeal rRNA genes (915aF, 5′‐AGGAATTGGCGGGGGAGCAC‐3′; 1386R, 5′‐GCGGTGTGTGCAAGGAGC‐3′) (Kittelmann et al. 2015). PCR amplifications were carried out in a total volume of 25 μL comprising 12.5 μL of 2× KAPATM HiFi HotStart ReadyMix (KAPA Biosystems, Wilmington, MA, USA), 0.75 μL of each primer (10 μmol/L), 10 μL of dH_2_O, and 1 μL of template DNA (5 ng/μL). The PCR conditions for bacteria were as follows: initial denaturation at 95°C for 5 min, followed by 30 cycles of denaturation at 98°C for 20 s, annealing at 55°C for 15 s, extension at 72°C for 15 s, and final extension at 72°C for 5 min. The PCR conditions for archaea were as follows: initial denaturation at 95°C for 5 min, followed by 28 cycles of denaturation at 98°C for 20 s, annealing at 59°C for 15 s, extension at 72°C for 15 s, and final extension at 72°C for 5 min.

#### 2.5.2 Bioinformatic Analysis

Raw sequencing reads were processed using the QIIME2 version 2021.4 (Bolyen et al. 2019). Primer sequences were removed from the reads using Cutadapt (Martin 2011). Sequence processing, such as denoising, quality filtering, dereplication, chimera removal, and merging, was conducted using the DADA2 plugin (Callahan et al. 2016). The exported amplicon sequence variants (ASVs) were rarefied to the lowest sequence depth for the diversity analysis. The bacterial and archaeal ASVs were classified phylogenetically using SILVA database version 138.99 (Quast et al. 2013) and the rumen and intestinal methanogen database (Seedorf et al. 2014), respectively. Furthermore, the closest taxonomy of ASVs was confirmed using BLAST against the NCBIref database. Sequence data were deposited in the DNA Data Bank of Japan sequence read archive under the accession number PRJDB35449 (DRR698133‐DRR698168).

### 2.6 Data Processing and Statistical Analysis

Eighteen experimental cows were evenly divided (*n* = 6) into low CH_4_:CO_2_ (LEm), mid‐CH_4_:CO_2_ (MEm), and high CH_4_:CO_2_ (HEm) groups based on their CH_4_:CO_2_ ratios in ascending order. Summarized data were statistically analyzed using fit model analysis of JMP software (version 17.0.0; SAS Institute Inc.). The differences in daily DMI, milk production parameters, CH_4_ production parameters, and daily eating and rumination times among the three groups were analyzed with the group as a fixed effect. In vitro incubation data were analyzed similarly. Because of the temporal isolation of one cow and inconsistent observations, daily DMI, eating and rumination time, and milk fatty acids and urea concentration were not recorded for the cow in the LEm group (*n* = 5). Because of the limited number of feeders recording feed weight, only 12 out of 18 cows (HEm: *n* = 4, MEm: *n* = 3, and LEm: *n* = 5) were used to analyze the diurnal changes in DMI and eating rate of the PMR at different time periods. Data on DMI, eating time, and eating rate at different time periods were analyzed as repeated measures, with time period, cow groups, and the interaction between time period and cow groups regarded as fixed effects, and cow nested within cow groups as random effects. Multiple comparisons among cow groups for these parameters were tested using Tukey's HSD test. Data were presented as least square means. Statistical differences in alpha diversity and microbial abundance among the cow groups were analyzed according to the multiple comparisons using the Wilcoxon rank sum test with Bonferroni adjustment. Statistically significant differences were declared at *p* < 0.05, whereas treatments with 0.05 ≤ *p* ≤ 0.10 were considered a trend toward significance.

## 3 Results

### 3.1 Methane Production, Feed Intake, Rumen Fermentation, and Milk Production

#### 3.1.1 Methane Production

Table 2 summarizes the variations in estimated CH_4_ production parameters among HEm, MEm, and LEm cows. The average parity (±standard error) was 2.0 ± 0.26, 2.3 ± 0.21, and 1.7 ± 0.21 for HEm, MEm, and LEm groups, respectively. Average days in milk at the first days of data collection were 198 ± 42.7, 221 ± 43.3, and 126 ± 43.7, respectively (Table 3). The CH_4_:CO_2_ ratio from breath samples exhibited significant differences among the cow groups (*p* < 0.01), with a 22.4% difference between HEm and LEm cows. Daily CH_4_ production was 29.6% lower (*p* < 0.05) in LEm cows than in HEm cows. Methane yield and intensity (CH_4_ production per unit of DMI and CH_4_ production per unit of ECM, respectively) were 20.7% and 20.1% lower (*p* < 0.05) in LEm cows than in HEm cows. The MCF was also lower (*p* < 0.05) by 14.9% in LEm cows than in HEm cows.

**TABLE 2:** Comparison in methane production parameters between divergent methane‐emitting dairy cows.

Item	Methane‐emitting group			SEM	*p*
	HEm	MEm	LEm		
CH_4_:CO_2_ ratio^2^	0.0861^a^	0.0776^b^	0.0668^c^	0.002	< 0.001
CH_4_ production, L/day^2^	644^a^	590^ab^	486^b^	30.3	0.007
CH_4_/DMI, L/kg^1^	24.7^a^	20.8^ab^	19.6^b^	1.15	0.023
CH_4_/ECM, L/kg^2^	15.9^a^	15.6^ab^	12.7^a^	0.85	0.034
Methane conversion factor, J/100 J^2^	5.21^a^	4.97^ab^	4.43^b^	0.17	0.019

Abbreviations: CH_4_:CO_2_, the ratio of CH_4_ to CO_2_ in breath gas collected by a sniffer method; DMI, dry matter intake; HEm, high CH_4_:CO_2_ ratio; LEm, low CH_4_:CO_2_ ratio; MEm, mid‐CH_4_:CO_2_ ratio; Methane conversion factor, estimated methane energy excretion/gross energy intake; SEM, standard error of means.

^1^ Observation number, HEm: *n* = 6, MEm: *n* = 6, LEm: *n* = 5.

^2^ Observation number, HEm: *n* = 6, MEm: *n* = 6, LEm: *n* = 6.

^a, b^Values with different superscripts within the same row differ significantly (*p* < 0.05).

**TABLE 3:** Comparison in body weight, feed intake, and eating and rumination behavior between divergent methane‐emitting dairy cows.

Item	Methane‐emitting group			SEM	*p*
	HEm	MEm	LEm		
Body weight, kg^2^	652^ab^	696^a^	607^b^	21.8	0.035
Parity^2^	2.0	2.3	1.7	0.23	0.153
Days in milk, day^2^	198	221	126	43.2	0.302
Visiting frequency to AMS, no./day^2^					
Milking	2.8	2.9	3.3	0.26	0.395
Feeding only	4.8	6.4	6.0	1.29	0.669
Dry matter intake^1^					
Partial mixed ration (PMR), kg/day	21.4	23.3	19.4	1.68	0.322
Concentrate at AMS, kg/day	5.14	5.49	6.53	0.47	0.160
Total, kg/day	26.6	28.8	25.9	1.94	0.580
Total per body weight, %	4.12	4.17	4.17	0.33	0.990
Eating time of PMR, min/day^1^	170	186	217	12.6	0.070
Eating rate of PMR, min/kg^1^ ^,^ ^3^	8.1^b^	8.1^b^	11.5^a^	0.62	< 0.003
Rumination time, min/day^1^	528	499	598	34.6	0.178
Estimated composition of consumed feed, % of dry matter^1^					
Concentrate diets	53.0^b^	52.7^b^	56.5^a^	0.82	0.014
Neutral detergent fiber	33.6^a^	33.7^a^	32.6^b^	0.23	0.014

Abbreviations: HEm, high CH_4_:CO_2_ ratio; LEm, low CH_4_:CO_2_ ratio; MEm, mid‐CH4:CO_2_ ratio; SEM, standard error of means.

^1^ Observation number, HEm: *n* = 6, MEm: *n* = 6, LEm: *n* = 5.

^2^ Observation number, HEm: *n* = 6, MEm: *n* = 6, LEm: *n* = 6.

^3^ Calculated by dividing eating time of PMR (min/day) by PMR intake (kg/day).

^a, b^Values with different superscripts within the same row differ significantly (*p* < 0.05).

#### 3.1.2 Feed Intake, Eating Pattern, and Body Weight

Feed intake and behavioral differences among HEm, MEm, and LEm cows are summarized in Table 3. No significant difference was observed among the three cow groups regarding daily DMI of PMR, concentrate diet provided at AMS, and total diets. Frequencies of visiting AMS with milking and feeding only were not different among cow groups. LEm cows had the lightest body weight (group effect, *p* = 0.04) among the three groups, with a significant difference (*p* < 0.05) between LEm and MEm cows. However, the relative DMI per body weight was not different among cow groups. Additionally, LEm cows tended (group effect, *p* = 0.07) to have a longer daily eating time of PMR than MEm and HEm cows. Eating rate of PMR (eating time per DMI of PMR) was also higher (*p* < 0.05) for LEm cows than those for MEm and HEm cows. However, the daily rumination time did not differ between the groups.

Based on the intake of PMR and concentrate diet provided at the milking robot (Table 3) and their feed composition (Table 1), the estimated compositions of total ingested diets were presented in Table 3. Because of the slightly but nonsignificantly greater intake of the concentrate diet at the milking robot, the proportion of concentrate diets was higher (*p* < 0.05), but NDF content was lower (*p* < 0.05) for the total diet consumed by LEm cows than those consumed by MEm and HEm cows.

Table 4 compares diurnal eating patterns among the three cow groups at 4‐h intervals. No significant differences were observed between the groups regarding DM intake of PMR at different time periods. All groups exhibited greater PMR intake at each time period between 1000 and 2200 h (time effect, *p* < 0.01). MEm cows tended to spend less time eating between 1000 and 2200 h compared with HEm and LEm groups. In contrast, LEm cows showed a tendency for longer eating times (group effect, *p* = 0.10) between 0200 and 1000 h compared with MEm and HEm cows. Although MEm and HEm cows exhibited a sudden shift toward longer eating times after 1000 h, the interaction effect was not significant.

**TABLE 4:** Comparison in eating behavior at 4‐h time intervals between divergent methane‐emitting dairy cows.

	Time period							*p*‐value		
Item	2–6 h	6–10 h	10–14 h	14–18 h	18–22 h	22–2 h	SEM	Group	Time	Interaction
PMR intake, kg DM^1^										
HEm	0.23	1.06	8.30	6.40	5.06	1.43	0.810	0.371	< 0.001	0.395
MEm	0.66	0.55	6.36	6.18	5.22	1.95	0.935			
LEm	1.55	1.37	5.74	4.54	4.03	2.02	0.724			
Average	0.81^b^	0.99^b^	6.80^a^	5.71^a^	4.77^a^	1.80^b^	0.478			
Eating time, min^1^										
HEm	3.6	12.8	51.3	48.2	43.8	12.6	6.61	0.102	< 0.001	0.813
MEm	6.9	8.6	45.2	47.8	42.9	28.1	7.63			
LEm	23.4	19.1	54.0	48.3	47.4	25.1	5.91			
Average	11.3^b^	13.5^b^	50.2^a^	48.1^a^	44.7^a^	22.0^b^	3.90			

Abbreviations: Group, the effect of groups; HEm, high CH_4_:CO_2_ ratio; Interaction, an interaction effect between group and time period; LEm, low CH_4_:CO_2_ ratio; MEm, mid‐CH4:CO_2_ ratio; PMR, partial mixed ration; SEM, standard error of means; Time, the effect of time period.

^1^ Observation number, HEm: *n* = 4, MEm: *n* = 3, LEm: *n* = 5.

^a, b^Values with different superscripts within the same row differ significantly (*p* < 0.05).

#### 3.1.3 Rumen Fermentation

Table 5 illustrates the differences in rumen fermentation profiles and salivary pH among HEm, MEm, and LEm cows. Salivary pH tended to be higher (group effect, *p* = 0.09) in LEm cows than in MEm and HEm cows. Rumen pH and total VFA concentrations showed no significant differences among the cow groups. However, LEm cows exhibited higher (*p* < 0.05) rumen ORP values compared with MEm cows. Additionally, LEm cows displayed a lower (*p* < 0.05) proportion of acetic acid and butyric acid and a higher (*p* < 0.05) proportion of propionic acid, resulting in a lower (*p* < 0.05) acetate‐to‐propionate ratio compared with HEm cows. The proportion of n‐valeric acid tended to be higher in LEm cows than in HEm cows (group effect, *p* = 0.09).

**TABLE 5:** Comparison in salivary pH and rumen fermentation parameters between divergent methane‐emitting dairy cows.

Item	Methane‐emitting group			SEM	*p*
	HEm	MEm	LEm		
pH					
Saliva	8.73	8.82	8.89	0.045	0.087
Rumen	6.62	6.59	6.48	0.110	0.661
ORP (mV)	−328^ab^	−382^b^	−313^a^	16.55	0.024
Total VFA (mmol/L)	123	125	121	4.220	0.728
VFA proportion (mol/100 mol)					
Acetic acid	66.7^a^	64.5^ab^	62.5^b^	0.693	0.003
Propionic acid	19.1^b^	21.6^b^	25.6^a^	0.912	0.001
n‐Butyric acid	11.3^a^	10.9^a^	8.70^b^	0.563	0.012
Iso‐butyric acid	0.68	0.68	0.60	0.055	0.543
n‐Valeric acid	1.17	1.35	1.47	0.087	0.089
Iso‐valeric acid	1.14	1.01	1.16	0.122	0.636
Acetate: propionate	3.54^a^	3.03^ab^	2.46^b^	0.158	0.001

Abbreviations: HEm, high CH_4_:CO_2_ ratio; LEm, low CH_4_:CO_2_ ratio; MEm, mid‐CH_4_:CO_2_ ratio; ORP, oxidation–reduction potential; SEM, standard error of means; VFA, volatile fatty acid.

^a, b^Values with different superscripts within the same row differ significantly (*p* < 0.05).

#### 3.1.4 Milk Production

No significant difference was observed in daily milk yield, ECM yield, and milk composition among the three groups (Table 6). For the milk fatty acid composition, the proportion of butyric acid (C4:0), caproic acid (C6:0), caprylic acid (C8:0), palmitoleic acid (C16:1), gamma‐linolenic acid (C18:3n6), and alpha‐linolenic acid (C18:3n3) was lower (*p* ≤ 0.05) in LEm cows than in HEm and MEm cows. Conversely, palmitic acid (C16:0) was higher in LEm cows than in MEm cows (*p* < 0.05). Additionally, the proportion of C20:0 was higher (*p* < 0.05) in LEm cows than in HEm cows.

**TABLE 6:** Comparison in milk yield and composition between divergent methane‐emitting dairy cows.

Item	Methane‐emitting group			SEM	*p*
	HEm	MEm	LEm		
Milk yield, kg/day^2^	41.6	38.5	42.6	3.66	0.709
Energy‐corrected milk (kg/day)^2^	42.1	38.0	38.1	3.09	0.582
Milk composition, %^2^					
Fat	4.05	4.07	3.27	0.33	0.179
Protein	3.35	3.34	3.12	0.16	0.492
Solid nonfat	8.87	8.86	8.73	0.13	0.723
Lactose	4.51	4.51	4.64	0.05	0.174
Somatic cell count, 10^4^/mL^2^	9.21	7.04	8.50	3.08	0.881
Milk urea nitrogen, mg/dL^1^	10.6	10.8	11.5	0.54	0.500
Fatty acid composition, % of total fatty acid^1^					
C4:0	2.44^a^	2.46^a^	2.08^b^	0.07	0.005
C6:0	1.93^a^	1.95^a^	1.46^b^	0.08	0.002
C8:0	1.28^a^	1.29^a^	0.99^b^	0.07	0.016
C10	3.03	3.15	2.58	0.20	0.163
C12:0	3.44	3.50	3.02	0.23	0.334
C14:0	12.2	11.3	12.0	0.44	0.392
C14:1	1.02^b^	0.80^b^	1.30^a^	0.07	0.001
C16:0	36.8^ab^	35.4^b^	38.5^a^	0.72	0.035
C16:1	1.54	1.80^a^	1.15^b^	0.10	0.002
C18:0	11.0	12.2	11.5	0.66	0.466
C18:1	20.8	21.5	20.6	1.13	0.853
C18:2	3.64	3.50	3.61	0.08	0.461
C18:3n3	0.29^a^	0.30^a^	0.22^b^	0.02	0.018
C18:3n6	0.04^ab^	0.04^a^	0.03^b^	0.003	0.034
C20:0	0.11^b^	0.13^ab^	0.15^a^	0.01	0.020
C20:4n6	0.13	0.11	0.10	0.01	0.290

Abbreviations: HEm, high CH_4_:CO_2_ ratio (*n* = 6); LEm, low CH_4_:CO_2_ ratio (*n* = 5); MEm, mid‐CH_4_:CO_2_ ratio (*n* = 6); SEM, standard error of means.

^1^ Observation number, HEm: *n* = 6, MEm: *n* = 6, LEm: *n* = 5.

^2^ Observation number, HEm: *n* = 6, MEm: *n* = 6, LEm: *n* = 6.

^a, b^Values with different superscripts within the same row differ significantly (*p* < 0.05).

#### 3.1.5 In Vitro VFA and Methane Production Incubated With Saliva From Individual Cows

Table 7 presents the results of in vitro ruminal VFA and CH_4_ production after incubation with saliva from HEm, MEm, and LEm cows, along with these parameters obtained from incubation with the buffer. Total gas and CH_4_ production did not differ significantly among the cow groups. Similarly, there were no differences observed in total VFA production across the groups, and VFA proportions remained consistent regardless of incubation with saliva from the three groups.

**TABLE 7:** Comparison in in vitro ruminal fermentation and gas production after diet incubation with McDougall's buffer and saliva obtained from divergent methane‐emitting dairy cows.

Item		Methane‐emitting group				
	Buffer	HEm	MEm	LEm	SEM	*p*‐value
pH	6.51 ± 0.01	6.41	6.37	6.41	0.029	0.422
Gas production (10^2^/mL)						
Total gas	6.70 ± 0.04	6.30	6.23	6.15	0.119	0.680
CH_4_	0.41 ± 0.06	0.41	0.38	0.42	0.211	0.354
Total VFA (mmol/L)	110 ± 0.06	119	115	120	6.484	0.867
VFA proportion (mol/100 mol)						
Acetic acid	49.1 ± 0.04	48.1	42.3	47.7	2.590	0.230
Propionic acid	20.2 ± 0.06	25.1	28.3	25.7	1.950	0.470
n‐Butyric acid	17.9 ± 0.02	15.5	17.3	14.9	1.112	0.297
Iso‐butyric acid	2.95 ± 0.02	2.77	3.20	2.93	0.221	0.381
n‐Valeric acid	4.93 ± 0.03	4.56	4.71	4.76	0.289	0.870
Iso‐valeric acid	4.95 ± 0.02	4.05	4.16	4.00	0.263	0.916
Acetate: propionate	4.06 ± 0.01	2.11	1.74	1.98	0.275	0.637

*Note:* Values represent simple averages of triplicate runs (Buffer) with standard errors and least‐square means for HEm, MEm, and LEm.

Abbreviations: Buffer, incubation with McDougall's buffer (*n* = 3); HEm, incubation with saliva from high CH_4_:CO_2_ ratio cows (*n* = 6); LEm, incubation with saliva from low CH_4_:CO_2_ ratio cows (*n* = 5); MEm, incubation with saliva from mid‐CH_4_:CO_2_ ratio cows (*n* = 6); SEM, standard error of means; T, treatment effect; VFA, volatile fatty acid.

### 3.2 Rumen Microbial Population

#### 3.2.1 Diversity of the Rumen Microbial Community

Table 8 summarizes the alpha diversity indices (Shannon index, Simpson index, Richness, ACE, and Chao1) of the rumen microbial community in HEm, MEm, and LEm cows. Bacterial diversity was lower in LEm than in HEm and MEm groups. However, no significant difference was observed in Shannon and Simpson indices for archaea among the cow groups.

**TABLE 8:** Alpha diversity of rumen bacterial and archaeal community from divergent methane‐emitting dairy cows.

	Methane‐emitting groups		
	HEm	MEm	LEm
Bacteria			
Shannon	6.06 ± 0.07	5.98 ± 0.04	5.14 ± 0.15
Simpson	0.99 ± 0.001	0.99 ± 0.0003	0.98 ± 0.003
Richness	892 ± 42.45	872 ± 31.23	560 ± 42.97
ACE	895 ± 42.67	875 ± 31.34	561 ± 43.07
Chao1	897 ± 42.60	877 ± 31.11	561 ± 43.15
Archaea			
Shannon	2.04 ± 0.13	2.11 ± 0.11	1.37 ± 0.16
Simpson	0.79 ± 0.03	0.79 ± 0.03	0.55 ± 0.06
Richness	26.0 ± 1.33	26.7 ± 1.74	23.3 ± 2.50
ACE	26.4 ± 1.46	26.7 ± 1.78	23.6 ± 2.62
Chao1	26.1 ± 1.38	26.7 ± 1.74	23.7 ± 2.76

*Note:* Values were means with standard errors.

Abbreviations: HEm, high CH_4_:CO_2_ ratio (*n* = 6); LEm, low CH_4_:CO_2_ ratio (*n* = 6); MEm, mid‐CH_4_:CO_2_ ratio (*n* = 6).

#### 3.2.2 The Composition of the Rumen Bacterial and Archaeal Community

The composition of bacterial and archaeal communities was examined to explore differences in rumen microorganisms among methane‐emitting groups. At the phylum level, *Firmicutes* were predominant (HEm: 42.8%, MEm: 44.3%, LEm: 53.4%), followed by *Bacteroidota* (HEm: 49%, MEm: 48.3%, LEm: 38%), *Actinobacteriota* (HEm: 2.47%, MEm: 2.65%, LEm: 4.32%), and *Patescibacteria* (consistent at 3% across groups). *Euryarchaeota* dominated archaeal communities at the phylum level. At the family level, *Prevotellaceae* were prominent (*p* < 0.05) in MEm and HEm (37.5% and 37.3%, respectively), whereas *Lachnospiraceae* were more abundant in LEm (21.6%) than in MEm (16.2%, *p* < 0.10) and HEm (13.4%, *p* < 0.05).

**FIGURE 1:**
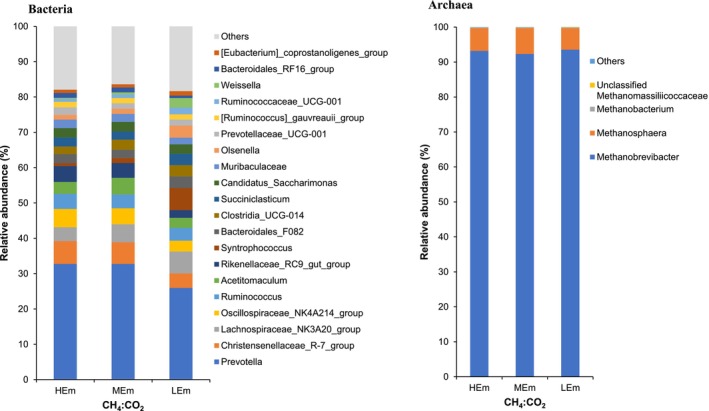
Taxonomic composition of rumen bacterial and archaeal community at the genus level between divergent CH_4_:CO_2_ ratio dairy cows; HEm, high CH_4_:CO_2_ ratio (*n* = 6); MEm, mid‐CH_4_:CO_2_ ratio (*n* = 6); and LEm, low CH_4_:CO_2_ ratio (*n* = 6). The top 20 bacterial and top 4 archaeal genera were shown, and the others were included in “Others.”

At the genus level (Figure 1), *Prevotella* showed the most predominant composition, being more abundant in HEm (32.7%, *p* < 0.05) and MEm (32.8%, *p* < 0.10) than in LEm (26.0%), followed by *Lachnospiraceae_NK3A20_group* (average: 5.06%), *Christensenellaceae_R‐7_group* (average: 5.57%), and *Oscillospiraceae_NK4A214_group* (average: 4.26%). *Rikenellaceae_RC9_gut_group* and *Oscillospiraceae_NK4A214_group* were also abundant (*p* < 0.05) in HEm and MEm than in LEm. Conversely, *Syntrophococcus* was more abundant in LEm (6.26%) than in MEm (1.44%, *p* < 0.10) and HEm (0.88%, *p* < 0.05). *Methanobrevibacter* was the dominant archaeal genus across all groups (LEm: 93.6%, MEm: 92.3%, HEm: 93.2%).

#### 3.2.3 Taxonomy of Rumen Microorganisms Associated With Methane‐Emitting Groups

The taxonomy of bacterial and archaeal ASVs was analyzed to elucidate rumen microorganisms linked to low CH_4_ emission in cows. For the top 10 bacterial ASVs (Table 9), ASVs attributed to *Bilifractor porci* (ASV1), *Weissella jogaejeotgali* (ASV10), and *Parafannyhessea umbonate* (ASV12) exhibited higher abundance (*p* < 0.05) in the LEm group than in the HEm group. Conversely, ASVs associated with *Xyalnibacter ruminicola* (ASV11) and *Prevotella micans* (ASV15) were more prevalent (*p* < 0.05) in the HEm group than in the LEm group. Other ASVs belonging to *Prevotellaceae* (ASV3, 6, 17, and 18 attributed to *X. ruminicola*) also showed the trend of abundance (*p* < 0.15) in HEm groups. For archaeal ASVs (Table 10), ASV1 belonging to *Methanobrevibacter olleyae* and ASV19 belonging to *Methanobrevibacter gottschalkii* were more abundant (*p* < 0.05) in the LEm group than in the HEm groups, whereas ASV2 belonging to *M. gottschalkii* (*p* < 0.05) and ASV4 of *M. ruminantium* (*p* < 0.10) were more abundant in the HEm group.

**TABLE 9:** Taxonomy of the dominant bacterial ASVs between divergent methane‐emitting dairy cows.

	BLAST hit			Relative abundance (%)			Fold change
ASV	Family	Closest species	Identity	HEm	MEm	LEm	log_2_(L/H)
ASV1	Lachnospiraceae	*Bilifractor porci*	95.0	0.657	1.266	6.041	3.20*
ASV2	Prevotellaceae	*Prevotella mizrahii*	99.8	0.742	0.129	4.585	2.63
ASV3	Prevotellaceae	*Xylanibacter ruminicola*	92.0	1.543	2.057	0.420	−1.88
ASV4	Prevotellaceae	*Xylanibacter ruminicola*	95.3	0.565	0.687	2.754	2.29
ASV5	Lachnospiraceae	*Blautia luti*	93.8	1.201	1.580	1.196	−0.01
ASV6	Prevotellaceae	*Xylanibacter ruminicola*	91.7	1.477	2.008	0.388	−1.93
ASV7	Prevotellaceae	*Xylanibacter ruminicola*	97.2	1.000	1.225	0.701	−0.51
ASV8	Oscillospiraceae	*Ruminococcus bromii*	95.8	0.330	0.164	2.221	2.75
ASV9	Lentimicrobiaceae	*Lentimicrobium saccharophilum*	86.1	0.568	0.450	1.685	1.57
ASV10	Lactobacillaceae	*Weissella jogaejeotgali*	100.0	0.231	0.483	1.987	3.10*
ASV11	Prevotellaceae	*Xylanibacter ruminicola*	94.8	1.295	1.220	0.124	−3.38*
ASV12	Atopobiaceae	*Parafannyhessea umbonata*	100.0	0.081	0.333	2.153	4.74*
ASV13	Eubacteriales incertae sedis	*Intestinimonas butyriciproducens*	93.1	0.989	0.990	0.478	−1.05
ASV14	Oscillospiraceae	*Ruminococcus bovis*	92.8	0.504	0.732	1.148	1.19
ASV15	Prevotellaceae	*Prevotella micans*	90.5	0.928	0.986	0.094	−3.30*
ASV16	Oscillospiraceae	*Ethanoligenens harbinense*	89.2	0.658	0.645	0.642	−0.04
ASV17	Prevotellaceae	*Xylanibacter ruminicola*	94.8	0.790	0.924	0.193	−2.04
ASV18	Prevotellaceae	*Xylanibacter ruminicola*	95.7	0.911	0.902	0.000	—
ASV19	Lachnospiraceae	*Faecalicatena orotica*	94.5	0.515	0.859	0.435	−0.24
ASV20	Eubacteriales incertae sedis	*Flintibacter butyricus*	94.6	0.674	0.516	0.592	−0.19

Abbreviations: ASVs, amplicon sequence variants; HEm, high CH_4_:CO_2_ ratio (*n* = 6); LEm, low CH_4_:CO_2_ ratio (*n* = 6); MEm, mid‐CH_4_:CO_2_ ratio (*n* = 6).

* Indicate a significant difference between HEm and LEm (*p* < 0.05).

**TABLE 10:** Taxonomy of the dominant archaea ASVs between divergent methane‐emitting dairy cows.

	BLAST hit			Relative abundance (%)			Fold change
ASV	Family	Closest species	Identity	HEm	MEm	LEm	log_2_(L/H)
ASV1	Methanobacteriaceae	*Methanobrevibacter olleyae*	98.0	28.213	20.901	62.905	1.157*
ASV2	Methanobacteriaceae	*Methanobrevibacter gottschalkii*	98.9	24.683	22.862	9.359	−1.399*
ASV3	Methanobacteriaceae	*Methanobrevibacter ruminantium*	99.1	7.498	11.231	3.071	−1.288
ASV4	Methanobacteriaceae	*Methanobrevibacter ruminantium*	98.9	9.988	6.963	1.208	−3.048
ASV5	Methanobacteriaceae	*Methanobrevibacter gottschalkii*	99.1	4.767	3.992	7.484	0.651
ASV6	Methanobacteriaceae	*Methanosphaera cuniculi*	95.6	5.195	5.294	5.244	0.013
ASV7	Methanobacteriaceae	*Methanobrevibacter ruminantium*	99.6	0.146	8.290	0.018	−3.044
ASV8	Methanobacteriaceae	*Methanobrevibacter gottschalkii*	98.7	3.142	1.799	0.788	−1.995
ASV9	Methanobacteriaceae	*Methanobrevibacter millerae*	98.7	1.883	1.715	1.261	−0.579
ASV10	Methanobacteriaceae	*Methanobrevibacter gottschalkii*	98.2	1.956	1.799	0.265	−2.884
ASV11	Methanobacteriaceae	*Methanosphaera cuniculi*	95.2	1.305	1.746	0.775	−0.752
ASV12	Methanobacteriaceae	*Methanobrevibacter ruminantium*	98.9	1.281	1.844	0.329	−1.961
ASV13	Methanobacteriaceae	*Methanobrevibacter gottschalkii*	98.9	0.956	0.976	1.391	0.541
ASV14	Methanobacteriaceae	*Methanobrevibacter millerae*	98.2	1.245	1.177	0.854	−0.543
ASV15	Methanobacteriaceae	*Methanobrevibacter gottschalkii*	98.5	1.543	0.788	0.316	−2.289
ASV16	Methanobacteriaceae	*Methanobrevibacter millerae*	98.5	0.766	0.998	0.715	−0.099
ASV17	Methanobacteriaceae	*Methanobrevibacter gottschalkii*	98.2	1.749	0.216	0.117	−3.901
ASV18	Methanobacteriaceae	*Methanobrevibacter gottschalkii*	98.2	0.824	0.751	0.241	−1.775
ASV19	Methanobacteriaceae	*Methanobrevibacter gottschalkii*	98.7	0.212	0.413	1.137	2.423*
ASV20	Methanobacteriaceae	*Methanobrevibacter ruminantium*	99.3	0.000	1.190	0.000	—

Abbreviations: ASVs, amplicon sequence variants; HEm, high CH_4_:CO_2_ ratio (*n* = 6); LEm, low CH_4_:CO_2_ ratio (*n* = 6); MEm, mid‐CH_4_:CO_2_ ratio (*n* = 6).

* Indicate a significant difference between HEm and LEm (*p* < 0.05).

## 4 Discussion

### 4.1 Methane Production and Eating Behavior

This study offers a comprehensive analysis of the physiological, behavioral, and dietary differences and their relation to the rumen microbial population among high, mid, and low CH_4_:CO_2_ ratio cows. For estimating the daily CH_4_ production of the individual cows, the milk production parameters (ECM and LW) and CH_4_:CO_2_ ratios in the breath measured at AMS by a sniffer method were applied to the estimating equation. Because of no differences in ECM yield among the three groups, variations in the estimated CH_4_ production were evidently affected by the CH_4_:CO_2_ ratio and LW; the daily CH_4_ production was notably higher in HEm than in LEm cows as expected. The CH_4_:CO_2_ ratio in respiration gas was suggested to be a useful proxy for evaluating CH_4_ production in cows with similar milk production levels (Uemoto et al. 2024). Suzuki et al. (2021) reported that the CH_4_ production of cows estimated with CH4:CO_2_ ratio measured by the sniffer method showed higher correlations with the value measured by a head box method.

In this study, variation in CH_4_:CO_2_ in the cows was associated with their body weight, with LEm cows weighing less than HEm and MEm cows. Live weight is phenotypically linked to CH_4_ production of cows (Herd et al. 2014). Furthermore, the daily eating rate of PMR was greater in LEm cows than in MEm and HEm cows, suggesting that eating behavior may affect the CH_4_:CO_2_ ratio and CH_4_ production of dairy cows. Previous studies have shown that eating rate (eating time per kg of DMI) negatively correlates with body weight (Dado and Allen 1994). A similar trend was also observed between different breeds (Holstein vs. Jersey cows) differing in body weight (Aikman et al. 2008). Smaller cows, with smaller rumen and mouth, may be limited in their bite size, necessitating more frequent bites and longer chewing times, resulting in faster food passage through the digestive system (Aikman et al. 2008). Digesta passage rate from the rumen is proposed as one of the factors influencing individual differences in CH_4_ production in ruminants (Cabezas‐Garcia et al. 2017). Okine et al. (1989) reported that increasing the passage rate by increasing rumen contraction reduced methane production from cattle. The passage rate of feed particles also increases with increased relative feed intake per BW (Krizsan et al. 2010). However, in this study, the statistical difference in DMI per BW between cow groups was not detected, similarly to the study of Aikman et al. (2008). Further study would be necessary to elucidate the relationship between feeding behavior, passage rate, and CH_4_ production of individual cows.

Ruminants spend a significant amount of time ruminating, and variation in rumination time is proposed as another factor influencing individual differences in daily CH_4_ production (Mikuła et al. 2022). However, in this study, no significant differences in daily rumination time were observed among LEm, MEm, and HEm cows, precluding an association of ruminating time with individual differences in CH_4_ production.

Mastication during eating and rumination stimulates salivary secretion, leading to variations in saliva secretion (Beauchemin 2018). Increased saliva secretion is associated with shorter mean retention times of liquid and small particles, which can reduce CH_4_ production (Zhang et al. 2023). Conversely, Palma‐Hidalgo et al. (2021) indicated that saliva stimulates rumen fermentation in vitro, even though it contains antibacterial compounds (Akula et al. 2023). Thus, it is hypothesized that differences in qualitative saliva properties such as concentrations of antimicrobial compounds among cows may affect rumen microbial populations and their fermentation processes. To test this hypothesis, we conducted in vitro gas production assays using saliva collected from individual cows. However, in this study, in vitro CH_4_ production did not differ among the cow groups. It is possible that the qualitative property of saliva collected via suction may not fully represent that secreted during eating and rumination. Further investigation is warranted to elucidate the specific effects of the qualitative property of secreted saliva on CH_4_ production.

Another important exogenous factor affecting methane production is dietary composition: providing high concentrate diets could reduce methane production in ruminants (Beauchemin et al. 2022). Slight but significantly higher levels of the concentrate diets and lower levels of NDF contents in daily rations consumed may be alternative reasons for the lower methane production in LEm cows. In AMS, the amount of concentrate diet is automatically decided and provided according to the level of milk production of each cow. Slightly higher milk production in LEm cows might result in a nonsignificantly but numerically greater provision of the concentrate diet.

### 4.2 Rumen Fermentation and Milk Component

This study suggests potential differences in rumen fermentation among cows exhibiting divergent CH_4_:CO_2_ ratios and estimated CH_4_ production, as well as their links to microbial communities. LEm cows exhibited a lower proportion of acetate and a higher proportion of propionate, leading to a decreased acetate‐to‐propionate ratio. This finding aligns with existing research indicating that a lower acetate‐to‐propionate ratio is associated with reduced CH_4_ production in ruminants (Janssen 2010; Stepanchenko et al. 2023). Additionally, an increasing trend in the concentration of valeric acid in LEm cows suggests reduced methanogenesis, as previously reported by Shabat et al. (2016).

Furthermore, differences in acetate and propionate proportions may also be associated with differences in milk fatty acid profiles among the groups. For milk fatty acid composition, lower ruminal acetate in LEm cows might result in lower C4:0, C6:0, and C8:0 composition in milk, reflecting that these fatty acids are synthesized from acetate absorbed from the rumen. The report of meta‐analysis (van Lingen et al. 2014) showed that CH_4_ yield (CH_4_ production per DM intake) had positive relationships with milk C6:0, C8:0, and C10:0 composition.

### 4.3 Microbial Population

In this study, taxonomic profiling of rumen microorganisms was conducted to elucidate their association with CH_4_ production differences in cows. The findings revealed significant differences in the abundance of specific bacterial and archaeal ASVs between the low and high CH_4_:CO_2_ groups, partly supporting the observed differences in estimated CH_4_ production and ruminal VFA profiles among the cow groups in this study. ASVs attributed to *Bilifractor porci* (ASV1), *Weissella jogaejeotgali* (ASV10), and *Parafannyhessea umbonata* (ASV12) exhibited higher abundance in the LEm group than in the HEm group. These bacterial species produce lactate and succinate as the major fermentation products (Wylensek et al. 2020; Lee et al. 2015; Kraatz et al. 2011). The metabolic hydrogen is consumed during the propionate production pathway from intermediates such as succinate and lactate. Thus, propionate production competes with methanogens utilizing the metabolic hydrogen as a substrate of CH_4_ production and lowers enteric CH_4_ production (Moss et al. 2000). The higher abundance of lactate‐ and succinate‐producing bacteria in LEm cows may have enhanced propionate production from lactate and succinate, thereby decreasing CH_4_ production by hydrogenotrophic methanogens. We observed various *Prevotella* ASVs in both LEm and HEm groups. Danielsson et al. (2017) reported that several OTUs belonging to *Prevotella* species were more abundant in the low‐CH_4_ emitting cows than in the high‐CH_4_ emitting cows. *Prevotella* species have not only phylogenetic diversity but also genetic and functional differences, including end product profiles, that may associate with variation in CH_4_ production in cows (Bekele et al. 2010; Shinkai et al. 2024).

Regarding methanogenic archaea, ASV1 belonging to *M. olleyae* was more abundant in the LEm group, whereas ASV4 belonging to *M. ruminantium* was more abundant in the HEm group. These findings were partly inconsistent with previous observations that showed *M. ruminantium* was linked with lower CH_4_ production in dairy cows (Danielsson et al. 2012). The methanogenic species *M. olleyae* and *M. ruminantium* produce CH_4_ from hydrogen (Rea et al. 2007; Smith and Hungate 1958). *M. olleyae* can synthesize coenzyme M required for methyl‐transfer reaction during methanogenesis; however, *M. ruminantium M1* is unable to synthesize it (Kelly et al. 2016). Similar to *M. ruminantium M1*, *M. olleyae* encodes adhesin‐like proteins predicted to have a role in mediating interactions with other specific microorganisms (Kelly et al. 2016; Ng et al. 2016), which indicates that *M. olleyae* and *M. ruminantium* interact with specific members in the rumen microbial community of low and high‐CH_4_‐emitting cows, respectively.

Even though the factors contributing to the difference in the microbial composition between LEm and HEm cows described above were not clear, regarding the effects of feeding behaviors, in the study comparing Holstein and Jersey cows (De La Guardia‐Hidrogo and Paz 2021), the difference in microbial diversity was found between the two cow breeds, which have slightly different eating time feed intake behaviors. However, in another study with early lactation Holstein dairy cows, parameters of feeding behavior did not associate with particular ruminal bacterial groups (Schären et al. 2018). Regarding feed composition, microbial population and expression of related metabolic genes in the rumen of dairy cows were reported to be altered by forage‐to‐concentrate ratios (Zhang et al. 2020).

In conclusion, the findings of this study suggest that a combination of body size traits, eating behavior, feed composition, and specific bacteria species and their related fermentation processes are associated with the divergence of breath CH_4_:CO_2_ ratio and estimated CH_4_ production in dairy cows. Low CH_4_ emitters were lighter in body weight and displayed longer eating times compared with their high‐emitting counterparts and were confirmed to harbor specific bacteria species potentially beneficial for high propionate production. Furthermore, these findings reveal that the CH_4_:CO_2_ ratio in respiration gas or the prediction of CH_4_ production by the estimating equation with the ratio can be used for evaluating individual variation of CH_4_ production in dairy cows. Further research is warranted to elucidate the mechanisms of the interrelationship between eating behavior, rumen digestion dynamics, and specific microbial taxa for modulating CH_4_ production in dairy cows.

## Conflicts of Interest

The authors declare no conflicts of interest.
